# The Role of Atopy in COPD and Asthma

**DOI:** 10.3389/fmed.2021.674742

**Published:** 2021-08-18

**Authors:** Meropi Karakioulaki, Eleni Papakonstantinou, Antonios Goulas, Daiana Stolz

**Affiliations:** ^1^Clinic of Respiratory Medicine and Pulmonary Cell Research, University Hospital of Basel, Basel, Switzerland; ^2^First Laboratory of Pharmacology, Department of Medicine, School of Health Sciences, Aristotle University of Thessaloniki, Thessaloniki, Greece

**Keywords:** COPD, asthma, IgE, atopy, allergy

## Abstract

Common to several allergic diseases is the generation of immunoglobulin E (IgE) by plasma cells, when exposed to an innocuous antigen. Asthma and chronic obstructive pulmonary disease (COPD) are two prevalent chronic airway inflammatory diseases. Asthma is mediated in some patients through eosinophilic inflammatory mechanisms that include allergic sensitization and Th2-mediated immune airway response. COPD, on the other hand is mainly considered a Th1-mediated inflammatory process with neutrophilic predominance or a non-Th2 inflammation, occasionally associated with the presence of airway bacteria or viruses. IgE production appears to play an important role in the development of both COPD and asthma, as it has been associated to respiratory symptoms, lung function, bacterial and viral infections, airway remodeling and bronchial hyperreactivity in both diseases. The aim of this review is to summarize all current data concerning the role of specific and total IgE in COPD and asthma and to highlight similarities and differences in view of possible therapeutic interventions.

## Immunology of Asthma and COPD

T lymphocytes are distinguished by the presence of cell membrane molecules known as cluster of differentiation 4 (CD4) and CD8. The CD4 T-lymphocytes are also known as helper T cells and can be further subdivided into Th1 and Th2, producing, respectively, Th1- and Th2-type cytokines (1). Th1-type cytokines induce the proinflammatory response that leads to the elimination of intracellular parasites and to the propagation of autoimmune responses (1). The Th2-type cytokines, such as interleukins (IL)- 4, 5, and 13 are associated with the production of IgE and with the promotion of eosinophilic responses in atopy (1).

**Figure 1:**
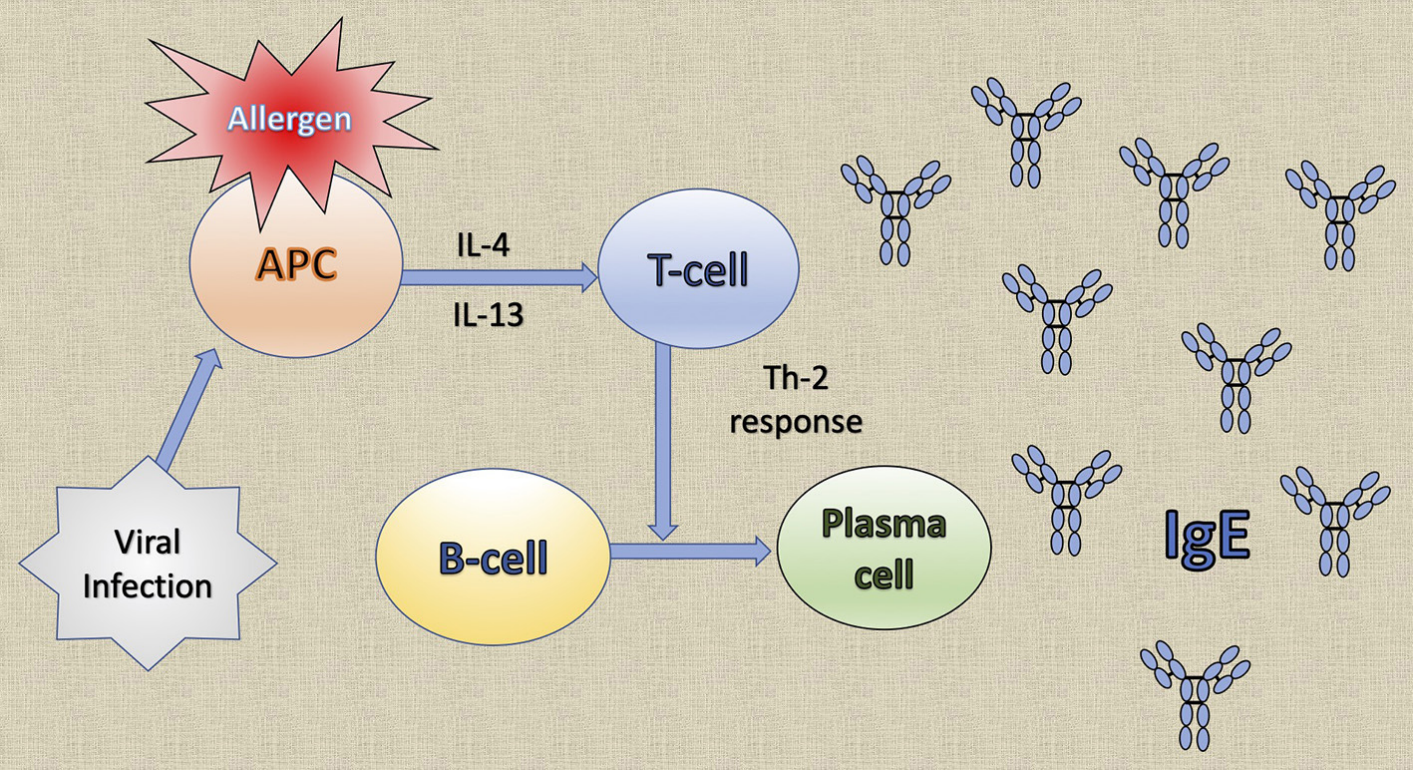
The generation of immunoglobulin E (IgE) by plasma cells. Upon initial allergen exposure, antigen presenting cells (APC) capture, process and present allergen peptides to T-cells. In the presence of interleukin (IL)-4 or IL-13, T-cells acquire the Th2 phenotype, proliferate and stimulate B-cells to differentiate into plasma cells that produce IgE. Viral infections induce the production of the T cell chemoattractant chemokine ligand 28 (CCL28) by dendritic cells and consequently recruit Th2 cells that lead to IgE production.

**Figure 2:**
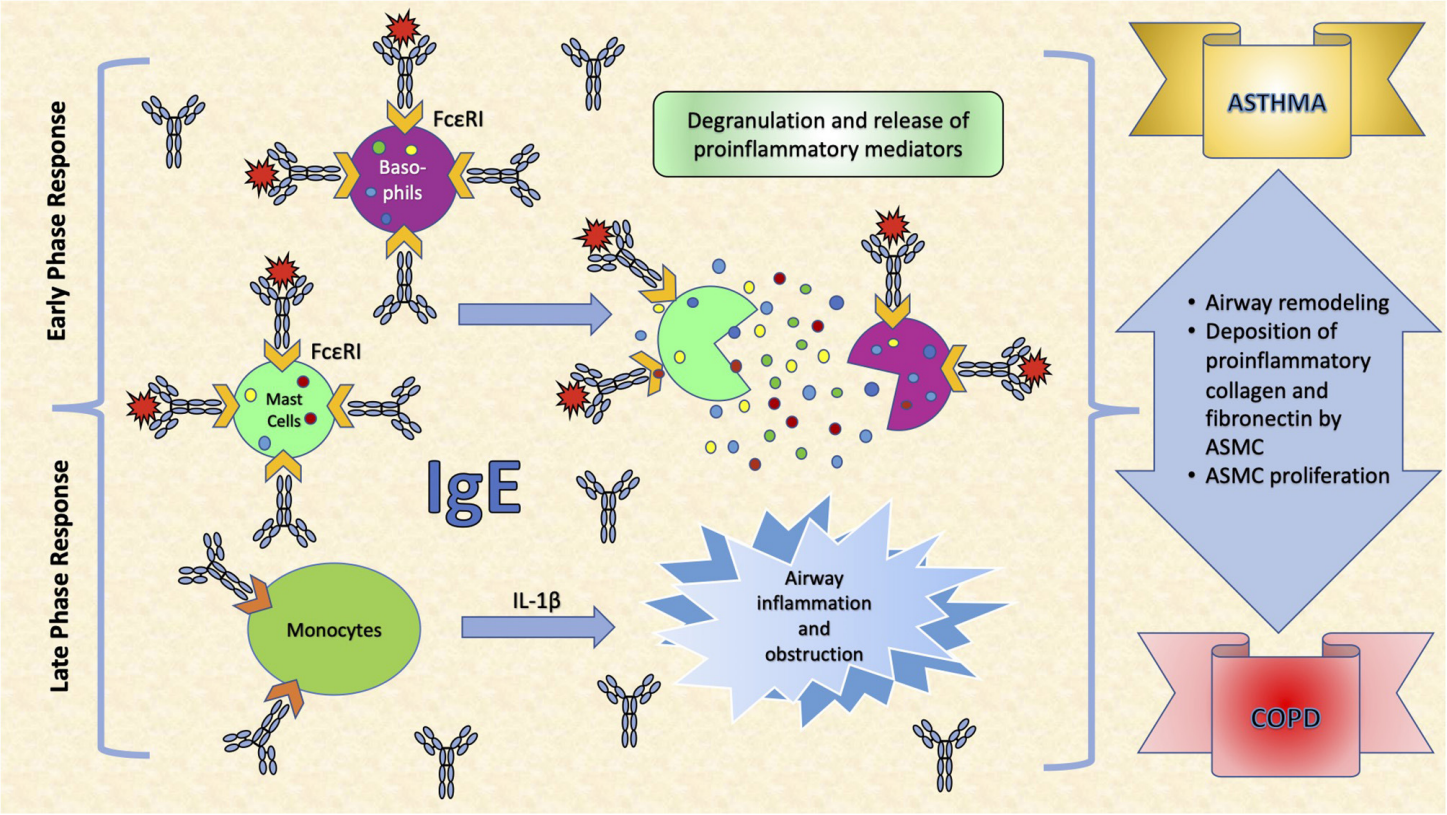
The effects of IgE in the respiratory system. IgE binds almost irreversibly to the high affinity IgE receptor (FcεRI) on the surface of mast cells or basophils to create allergen receptors. A subsequent exposure to the antigen leads to cross-linking of the IgE:FcεRI complex, degranulation of the cells and release of inflammatory mediators, leading to symptoms such as rhinorrhea, sneezing, nasal congestion and itching. Interleukin (IL)-1β is a very important pro-inflammatory cytokine that mediates the inflammatory response, and its expression may be induced in monocytes, as a response to increased IgE levels. This leads to airway inflammation and obstruction, an important determinant in COPD. Moreover, IgEs increase airway remodeling by increasing deposition of pro-inflammatory collagens and fibronectin by airway smooth muscle cells (ASMC) and stimulating their proliferation.

Common to several allergic diseases is the generation of immunoglobulin E (IgE) by plasma cells, when exposed to an innocuous antigen (2). Upon initial allergen exposure, antigen presenting cells capture, process and present allergen peptides to T-cells. In presence of IL-4 or IL-13, T-cells acquire the Th2-phenotype, proliferate and engage B-cells to differentiate into plasma cells, which produce IgEs (3) (Figure 1). IgEs bind almost irreversibly to the high affinity IgE receptor (FcεRI) on the surface of mast cells or basophils to create “allergen receptors” (2, 3). A subsequent exposure to the same antigen leads to cross-linking of the IgE:FcεRI complex, degranulation of the cells and release of inflammatory mediators, leading to the early phase response (EPR) (2, 3). EPR occurs immediately after exposure and is accompanied by symptoms such as rhinorrhea, sneezing, nasal congestion and itching (4). The ensuing effector cell infiltration into the tissue encompasses the late-phase response (LPR), along with the continuing production of IgE (4) (Figure 2).

Asthma and chronic obstructive pulmonary disease (COPD) are two common chronic airway inflammatory diseases. Asthma is often mediated through an eosinophilic

inflammatory mechanism that includes allergic sensitization and Th2-mediated immune airway responses (5). COPD, on the other hand is mainly considered a Th1-mediated inflammatory process with neutrophilic predominance or a non-Th2 inflammation that is occasionally associated with the presence of airway bacteria or viruses (6) (Table 1).

**Table 1:** Comparison of IgE production and the allergic profile in asthma and COPD patients.

**Asthma**	**COPD**
**DIFFERENCES**	
Mediated through eosinophilic inflammation and Th2-mediated inflammatory responses (5)	Mediated through neutrophilic inflammation and Th1-mediated inflammatory responses (6)
Total IgE levels were higher in 541 patients with self-report of doctor's diagnosis of asthma before the age of 40, compared to 598 controls without any airflow obstruction (7)	Total IgE levels among 899 patients with COPD were not significantly different when compared to the total IgE levels of 598 controls without any airflow obstruction (7)
Asthma patients tested for six specific IgEs against indoor aeroallergens were found to be more frequently positive at least to one specific IgE when compared to controls (49.9% of asthma patients vs. 30.3% of controls, *p* < 0.05) (7)	COPD patients tested for the six specific IgEs against indoor aeroallergens were not found to be more frequently positive at least to one specific IgE when compared to the controls (24.5% of COPD patients vs. 30.3% of controls, *p* > 0.05) (7)
**SIMILARITIES**	
Prevalence and profile of IgE-dependent sensitization to inhaled allergens is not different between asthma and COPD (8)	
Similar cytokine profiles in both asthma and COPD might indicate that both Th2 and Th1 cells are involved in the immunopathology of these diseases (8)	
Atopy and IgE-mediated sensitization to environmental allergens is strongly associated with asthma (9) and it increases the risk for COPD development (10, 11)	
Th2- inflammatory gene expression signature in COPD individuals without clinical history of asthma suggests shared mechanisms with asthma (12)	
Both allergic asthma and COPD are characterized by an overexpression of FcεRI on DCs (13–15)	
SE-IgE is associated with asthma severity, exacerbations, control and age of onset (16–21), as well as with COPD exacerbations and control (22)	
Airway hyperreactivity, a feature of asthma, can be an independent predictor of COPD development in the general population (23) and also a risk factor for rapid progression of airway obstruction in patients with mild COPD (24)	

*COPD, chronic obstructive pulmonary disease; S. aureus, Staphylococcus aureus; SE-IgE, staphylococcal enterotoxin IgE; ASMCs, airway smooth muscle cells; FcεRI, high affinity IgE receptor; DCs, dendritic cells*.

## Atopy in Asthma and COPD

IgE-mediated sensitivity of inhaled allergens is strongly associated with asthma (9), but this is not true for all asthma cases (8). Allergic asthma is the most common asthma phenotype, which is defined by the presence of allergic sensitization (25), or by a correlation between allergen exposure and asthmatic symptoms (26) but may overlap with other phenotypes (27). Patients with allergic asthma are more likely to report seasonal variations of their symptoms (25). Although it might present at any age, patients with allergic asthma are usually younger than those with non-allergic asthma (25, 26, 28, 29). In school-age children, aeroallergen sensitization is more frequent in children with severe asthma, rather than in children with mild-to-moderate asthma (30). In adults, allergic asthma has been associated with a greater FEV_1_ reversibility, a higher sputum eosinophil count, higher FENO levels and more exacerbations in the past year (31). Moreover, exercise-induced bronchospasm is more frequent and severe in allergic rather than in non-allergic asthma (32). Conversely, some studies suggest that allergic asthma is less severe than non-allergic (25, 26, 33–37), or cast doubt on the association between asthma severity and atopic status (38–41). Indeed, the different phenotypes of asthma are an important determinant in that matter, as the role of allergy differs in early onset asthma when compared to late onset asthma (42, 43). Accordingly, young asthmatics mostly present with allergic asthma, while late onset asthma is usually severe, steroid resistant and not related to allergy (42–44). Total serum IgE, peripheral eosinophilia levels, as well as Th2-type responses are usually higher in allergic vs. non-allergic asthma (27, 32, 45).

In a study of Bafadhel et al., atopy was defined as a positive skin prick test and/or elevated allergen specific IgEs and it showed a prevalence of 34% among COPD patients (46). Jin et al. showed that even among 273 COPD patients without obvious atopy, the prevalence of elevated total IgE was 47.3% (47). A meta-analysis by Putcha et al. indicated that 35% of the COPD patients (*N* = 403) from the SPIROMICS cohort and 36% of the COPD patients (*N* = 696) from the COPDGene cohort presented with atopy, defined as positive sensitization to any of the 10 indoor and outdoor allergens measured in the study (48). There was an almost 50% overlap between atopic status with COPD with asthmatic characteristics (defined as self-report of doctor diagnosed asthma in patients with COPD) in both cohorts (48). Moreover, COPD individuals with non-atopic asthmatic characteristics had the most impaired symptom scores (SGRQ = 4.2, 95% CI: 0.4–7.9; CAT score = 2.8, 95% CI: 0.089–5.4) and highest risk of exacerbations (incidence rate ratio = 1.41, 95% CI: 1.05–1.88), compared to the group without atopy or asthma, while COPD individuals with atopy and atopic asthma were not at increased risk of adverse outcomes (48).

A recent study in an Asian cohort recruited across three countries demonstrated that specific IgEs produced against a broad range of allergens (pollens, house dust mite, cockroach, and fungi) were increased in COPD (*n* = 466) when compared to controls (*n* = 51) (49). House dust mite (*B. tropicalis, D. pteronyssinus, D. farinae*) and grass pollens (pooids and panicoids) presented the highest specific IgE-binding intensities in COPD (49). A significant number of COPD patients demonstrated sensitization to fungi (*n* = 249, 55.8%) and house dust mites (*n* = 229, 51.3%) (49). Frequent exacerbators showed significantly increased specific IgE-binding to crude fungal allergens (*Curvularia, Penicillium, A. fumigatus*) and the cockroach allergen *Bl. germanica*, but no significant specific IgE-binding to pollens, house dust mites or the cockroach allergen *Pr. americana* (49). On the other hand, no association between sensitization status and the COPD GOLD stage (lung function) or GOLD group (ABCD) was detected, but a highly sensitized, fungal predominant subgroup of COPD patients demonstrated the worse clinical outcome, with greatest symptoms (median CAT score = 16, IQR = 10–22, *P* < 0.01), poorest lung function (FEV_1_ = 41.1% predicted, IQR = 32.5–57.0%, *p* < 0.01) and increased exacerbation rate (IRR 2.01, 95%CI = 1.44–2.81, *p* < 0.001) (49). Additionally, there was a significantly increased systemic IgE response to a number of outdoor air fungi (*Schizophyllum, p* < 0.01; *Aspergillus, p* < 0.001; *Penicillium, p* < 0.001; *Byssochlamys, p* < 0.001; and *Cladosporium, p* < 0.01) in COPD patients when compared to controls and an increased number of exacerbations was detected in the COPD patients that were sensitized against air-fungi (IRR = 2.29, 95%CI = 1.12–4.68, *p* < 0.01) (49). The study indicated that the abundance of indoor air and surface allergens positively correlates with COPD symptoms (*r* = 0.75, *p* < 0.01) and negatively with lung function (*r* = −0.61, *p* < 0.01), suggesting that indoor air and surfaces represent a potential source of fungal allergen exposure (49). However, blood eosinophil counts were not different among the high, moderate and low-sensitized patients (49). Notably, in this study patients were enrolled from Singapore, Hong Kong, and Malaysia (49). All these countries are located in South Asia where the temperature and humidity differ dramatically from other Asian and Western countries. It is important to note here that the prevalence and role of fungal sensitization in tropical climate countries may not be the same in temperate climate ones and further studies elucidating this difference should be conducted.

Total IgE serum levels have been previously associated with a longitudinal decline of lung function (FEV_1_/FVC), independent of smoking and asthma status (50) and therefore atopy is potentially associated with the risk for COPD (50). Several studies suggest that in COPD patients, IgE-mediated sensitization to environmental allergens plays an important role to the pathogenesis of the disease, as it has been associated with severe symptoms or deteriorating lung function (10, 11, 47, 51, 52). Fattahi et al. demonstrated that atopy in COPD was associated with a higher prevalence of cough and phlegm, but not with FEV_1_ decline (10). When compared to non-allergic controls, patients with increased serum total IgE have a more severe or longer history of respiratory symptoms, such as dyspnoea and a greater impairment of lung function (47, 51). Additionally, COPD individuals with allergic sensitization have been shown to have increased respiratory symptoms and exacerbation rates (52).

**Figure 3:**
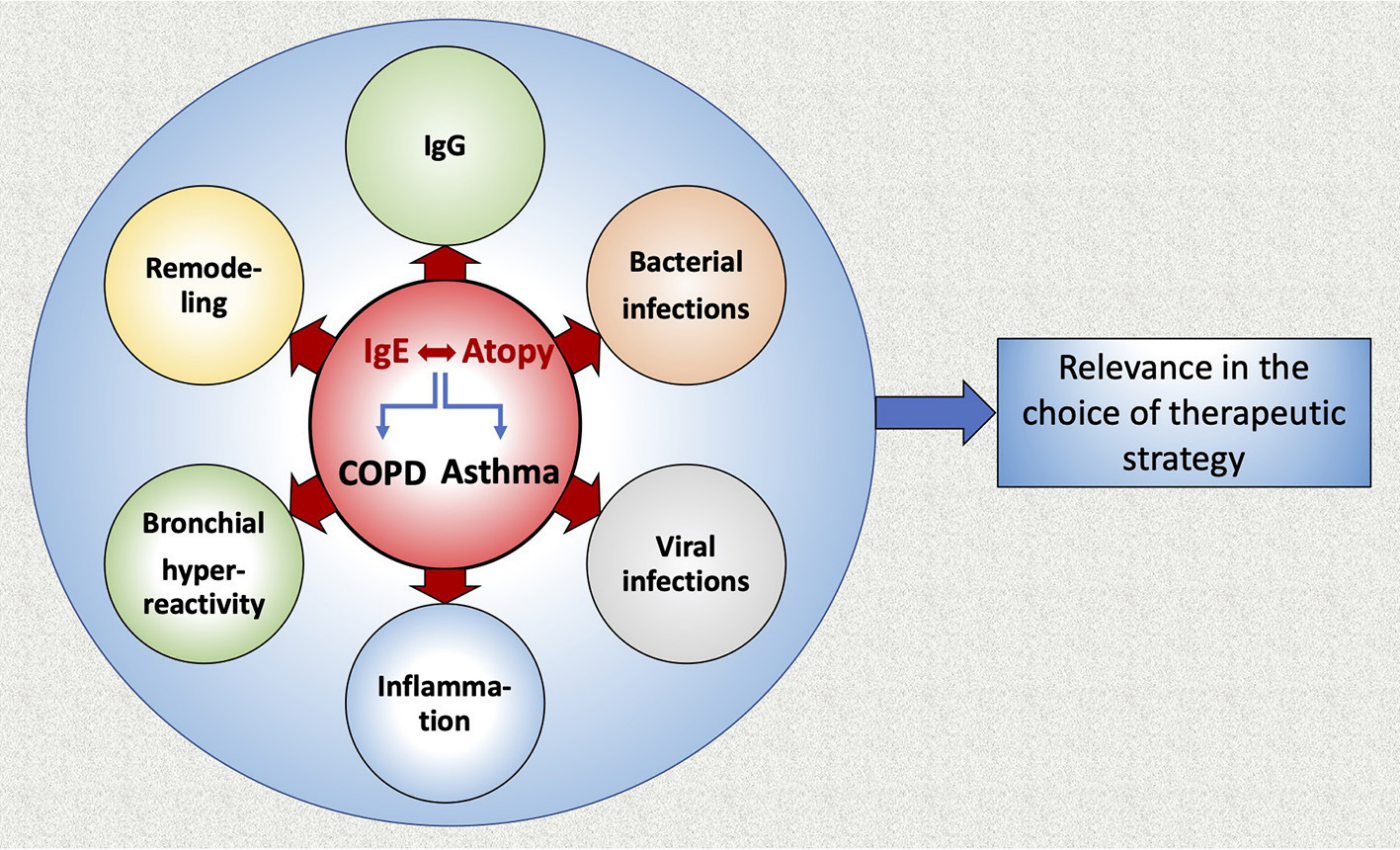
Graphic abstract of the review.

Another study indicated that in COPD, IL-1β and IgE serum levels correlate with clinical aspects of disease severity and suggested that the production of both IgE and IL-1β may be related to smoking, which affects airway obstruction (53). IL-1β is a very important pro-inflammatory cytokine that mediates the inflammatory response and its expression may be induced in monocytes, as a result of increased IgE levels (53) (Figure 3). However, in that study, no correlation between IL-1β and IgE was observed, probably due to the small sample size (30 COPD patients, 30 healthy controls) (53).

The Dutch hypothesis proposed in 1,961 states that there are common risk host factors for asthma and COPD, including airway hyperresponsiveness and atopy and that disease manifestations also depend on external factors, such as exposures (54). Indeed, the finding of a bronchial epithelial Th2- inflammatory gene expression signature in some COPD individuals suggests shared mechanisms with asthma (12). This implies that Th2-mediated airway responses may be important in COPD patients without clinical history of asthma (12). Bozek et al. demonstrated that neither the prevalence nor the profile of IgE-dependent sensitization to inhaled allergens differed between asthma and COPD (8). They pointed out that there are similar cytokine profiles in asthma and COPD and this could indicate that both Th2 and Th1 cells are involved in the immunopathology of these diseases (8).

Additionally, severe COPD and allergic asthma were characterized by a similar overexpression of FcεRI on plasmacytoid dendritic cells (DC) and this was in turn related to the reduction of asthma and COPD with asthmatic characteristics exacerbations following omalizumab treatment, as omalizumab suppresses this receptor (13–15). In view of the effect of anti-IgE therapies on asthma exacerbations, trials investigating the effect of anti-IgE on exacerbations of COPD without asthmatic characteristics are warranted. It appears that COPD patients display an overexpression of the FcεRI receptor on DC and this highlights the important role of IgE in the development and progression of COPD (55). Current smokers display an increased expression of the FcεRI receptor on myeloid and plasmacytoid DC, when compared to never smokers (55). Moreover, the overexpression of the FcεRI receptor on plasmacytoid DC is similar between patients with severe COPD and patients with allergic asthma, while in COPD patients it is associated with increased serum levels of total IgE, worse GOLD stage and worse lung function (55).

Hersh et al. reported that total IgE levels were higher in 541 patients with self-report of doctor's diagnosis of asthma before the age of 40, compared to 598 controls without any airflow obstruction (7). Moreover, when the asthma patients of that study were tested for six specific IgEs against indoor aeroallergens [cat and dog dander, dust mite (*D. farinae* and *D. pteronyssinus*), German cockroach and mold mix] they were found to be more frequently positive at least to one specific IgE when compared to controls (49.9% of asthma patients vs. 30.3% of controls, *p* < 0.05) (7). However, total IgE levels among 899 patients with COPD were not significantly different from total IgE levels of 598 controls without any airflow obstruction (7). Moreover, when COPD patients were tested for the same six specific IgEs against indoor aeroallergens, they were not found more frequently positive with regard to any IgE when compared to controls (24.5% of COPD patients vs. 30.3% of controls, *p* > 0.05). A higher proportion of current smokers had elevated total IgE levels and at least one positive specific IgE, when compared to former smokers (7). This is in agreement with the study of Omenaas et al., who reported potential effects of smoking on IgE levels (56).

## Interplay Between Atopy and Viral Infections in Asthma and COPD

Several observational studies have associated allergic sensitization by 1 year of age with respiratory viral infections in infancy (57–59), and severe respiratory infections in early age have been associated with a higher prevalence of asthma in later childhood (60, 61). The association between viral infections and allergic disease is not unreasonable, because the immune response against many viral infections includes the production of specific IgEs against viral pathogens (62–65). Therefore, anti-IgE therapy was shown to prevent viral exacerbations of asthma (66) and the exacerbation number was reduced even when omalizumab was given 4–6 weeks before children return to school in fall, especially in those who had recently experienced an asthma attack (67).

A viral infection can attract several cell types, such as Th1 cells, CD8 cells, neutrophils and DC, to the site of inflammation, and they can all participate in the allergic response of the patient (68) (Figure 1). Moreover, viral infections affect the expression of receptors involved in the allergic response (68) and can induce several inflammatory mediators, including TGF-β, neutrophilic elastase and several cytokines, which have an effect in the remodeling process of the lung (68). Thereby, they might produce long term effects on the structure of the developing lung, leading to suboptimal lung growth and function and increasing the risk of airway narrowing and the development of clinical asthma (68). That is why respiratory viral infections have similar symptoms with allergic diseases, like allergic rhinitis, and they clearly exacerbate asthma (69).

The etiology of IgE production during viral infections is not yet clearly understood, as IgE production is normally a result of Th2-biased responses (70). In severe viral infections, however, an antiviral Th1-biased response may lead to a proatopic Th2 response and the link between virus and atopy appears to be through the production of IgEs (71, 72). It has been demonstrated that IgEs are produced against a large number of human viral pathogens (73–80). Results from mouse model studies indicate that severe viral infections induce the production of antiviral IgEs that bind to the FcεRI receptor of DCs (71, 81–84). The receptor is then crosslinked by an antigen, the DCs produce the T cell chemoattractant chemokine ligand 28 (CCL28) and recruit IL-13-producing Th2 cells that can contribute to asthma (71, 81–84). CCL28 was found elevated in the lungs of patients with asthma (71, 85) (Figure 1).

Specifically, the Respiratory Syncytial Virus (RSV) was suggested to enhance Th2 sensitization to aeroallergens (86). Some studies have demonstrated that there is a correlation between the titer of antiviral IgEs and the severity of RSV symptoms, such as wheezing in infants and recurrent wheezing or development of asthma in older children (62, 64, 87). Moreover, infants requiring hospitalization for severe RSV infection in the first 6 months of life, have a nearly 20-fold increased risk of developing asthma (88).

Human rhinovirus (HRV) is also known to be a major cause of asthma exacerbations in infants and infection with HRV is associated with a higher incidence of asthma onset (61, 89–91). The prevalence of virus detection in adult asthma exacerbations was demonstrated in the range of 41–78% (92), and HRV appears to be a frequent cause of those exacerbations (93). Accordingly, in suburban children, from birth up to 3 years of age, who were at risk of asthma (one parent with asthma or allergy) and showed that the most important risk factor for development of wheeze by the third year of life was a symptomatic rhinovirus infection (94).

Children with parainfluenza infection in the first year of life had higher odds of developing asthma in their second year of life (95). In another study, infants younger than 3 months, infected with RSV, influenza or parainfluenza had comparable increases in their Th2 cytokine profiles, implicating that there are similarities between RNA respiratory viruses in their ability to push forward an atopic predisposition (96). A cohort of 90,000 children was examined and an increased risk of asthma was found in children with episodes of bronchiolitis during non-winter months (97). It was also found that patients born 4 months before the winter virus peak, had an increased risk of developing asthma, suggesting that the timing of the viral infection plays an important role in the development of early onset asthma (98).

A growing body of evidence implicates viral respiratory tract infections as the predominant risk factor associated with exacerbations of COPD and the development of chronic airway disease (99, 100). Most exacerbations of COPD are triggered by either bacterial or viral infections or a combination of both (101), since 40–80% of acute exacerbations of COPD (AECOPD) that frequently require hospitalization are attributed to viral respiratory tract infections (99). AECOPD associated with symptoms of a common cold have been shown to have a more sudden onset and longer recovery times than AECOPD without cold symptoms (102). Additionally, COPD patients having more frequent exacerbations experience nearly double the number of colds compared to patients experiencing fewer exacerbations (103) and the presence of cold symptoms is associated with a 15% risk of AECOPD (104). On the other hand, in a recent study, Stolz et al. have shown that the prevalence of viral infections during a stable period of COPD is low and that the risk of exacerbations following the onset of common cold symptoms depends on the particular virus associated with the event and is significant only for parainfluenza 3 (105).

## Interplay Between Atopy and Bacterial Infection in Asthma and COPD

There are a few reports regarding the relationship between asthma attacks and bacterial infections (106–109). Viral and bacterial infections were observed in 70% of inpatients with an asthma exacerbation in clinical practice, and infection with *Streptococcus pneumoniae* has been related to adult asthma exacerbation (110).

*Staphylococcous aureus* is a Gram+ coccus that colonizes humans, as well as domestic animals and is a common opportunistic pathogen (111). At least 20 serologically distinct staphylococcal superantigens have been described that include staphylococcal enterotoxins (SEs) A through V and toxic shock syndrome toxin-1 (TSST-1) (112, 113). Of the more than 20 staphylococcal enterotoxins, SEA and SEB are the best characterized and are also regarded as superantigens, because they are capable of binding to multiple types of the variable region on the beta chain of the T-cell receptor and stimulate large populations of T cells (polyclonal activation of T cells) (112, 114). The result of this massive T cell activation is a cytokine bolus leading to an acute toxic shock (115). Moreover, SEA and SEB induce polyclonal IgE formation (SEA-IgE and SEB-IgE), associated with allergic multi-morbidity in adolescents (116) and they may also activate B-cells, eosinophils, epithelial cells and others, resulting in a cytokine storm locally in the tissue and the generation of a strong inflammatory response (117).

*Staphylococcous aureus* is frequently found colonizing patients with Th2-biased diseases, such as atopic dermatitis and chronic rhinosinusitis with nasal polyps (118–122). It releases proteins that facilitate bacterial invasion and colonization and that exert immunosuppressive action on the mucosal environment (123–126). Asthmatic patients have increased IgE reactivity specific to various secreted *S. aureus* proteins (117). There is evidence that *S. aureus* is persistently colonizing the nasal mucosa of patients, protected by a biofilm or hiding inside immune cells (121) and is constantly producing a panel of factors that could initiate and aggravate a Th2-biased immune response (127). Chronic exposure to *S. aureus* secreted proteins could also hinder the resolution of inflammation, fostering chronification (127).

A number of studies have demonstrated in the past that sensitization to staphylococcal enterotoxins is associated with asthma severity (16–19), asthma exacerbations (17), asthma control and age of asthma onset (20, 21). There is only one study that associates increased SE-IgE to COPD exacerbations and COPD control but without defining the allergic profile of those COPD patients (22) (Table 1).

*Haemophilus influenza*e (NTHi) triggers histamine release through both IgE- and non-IgE-dependent mechanisms (128) from cells of the respiratory mucosa sensitized to the bacterium (129, 130). Specific anti-NTHi IgEs occur at a low level in healthy subjects, and patients with chronic bronchitis and moderate-severe COPD have elevated specific anti-NTHi IgEs compared to healthy controls, with higher anti-NTHi IgE levels in those with most severe disease. Additionally, specific anti-NTHi IgE levels are greater in those with moderate-severe COPD than in those with chronic bronchitis (131).

## Atopy and Airway Remodeling, Inflammation and Bronchial Hyperreactivity in Asthma and COPD

In allergic asthma, IgE increases airway remodeling by increasing deposition of pro-inflammatory collagens and fibronectin by airway smooth muscle cells (ASMC), as well as by stimulating their proliferation (132) (Figure 2). When ASMC are exposed *in vitro* to serum from allergic patients, their proliferation, as well as the deposition of collagen type-I (48 h) and of fibronectin (24 h) are stimulated (133). All these can be prevented by exposure of ASMC to allergic serum that is pre-incubated for 1 h with anti-IgE antibodies (omalizumab) (133).

Fang et al. have investigated the effect of IgE on human primary ASMC remodeling in the absence of allergens (non-immune IgE) *in vitro* (134). They reported that non-immune IgE (produced *in vitro* from a monoclonal hybridoma cell line and not induced by allergens) was sufficient to stimulate ASMC remodeling by upregulating microRNA-21-5p, which in turn downregulated phosphatase and tensin homolog (PTEN) expression and supported mammalian target of rapamycin (mTOR) signaling (134). Interestingly, they pointed out that the inhibition of microRNA-21-5p increased PTEN and reduced ASMC remodeling (134). Therefore, the suppression of microRNA-21-5p may present a therapeutic target to reduce airway wall remodeling (134).

Airway hyperreactivity is a general term to describe the increased response of the airways to bronchoconstricting agonists, such as methacholine or histamine, a hallmark of asthma (135). In COPD, the response to bronchoconstrictors is related to the degree of baseline airflow obstruction (136), in contrast to patients with asthma, without fixed airflow obstruction (23, 137, 138). There is an evident heterogeneity of remodeling in COPD patients in that the pathology may involve large or small airways (inflammation and increased wall-thickness), loss of small airways and emphysema (135). It is also recognized that COPD patients who have increased airway responsiveness are most commonly atopic and present with a higher degree of reversibility, as well as with eosinophilic airway inflammation, higher response to corticosteroids and faster decline in FEV_1_ (135). Moreover, airway hyperreactivity, can be an independent predictor of COPD development in the general population (139) and also a risk factor for rapid progression of airway obstruction in patients with mild COPD (24). However, it decreases after smoking cessation (140) (Table 1).

Remodeling of the airways is a well-recognized feature of COPD, depended on COPD severity based on post-bronchodilator FEV_1_ (141). There are several pathological changes in the lungs of COPD patients including: thickness of the airway wall and the airway smooth muscle layer (although not to the extent seen in asthma), increased blood vessel density, hypersecretion of mucus, metaplasia of the epithelial cells, enlargement of the submucosal glands, loss of terminal and respiratory bronchioles and enlargement and destruction of the alveoli, as well as neutrophilic inflammation and infiltration of CD8 T-lymphocytes (142–144). However, the effect of atopy on airway remodeling in COPD patients has not been studied yet. Understanding better this issue is of clinical importance, as it may assist us to apply the proper medical intervention for atopic and non-atopic COPD patients.

## Interplay Between IGG and IGE-Mediated Inflammatory Responses

A recent study demonstrated that IgG antibodies play a key role in controlling IgE-mediated inflammatory responses in patients with nasal polyps, by interfering with allergens potentially binding to cell-bound IgEs (145). Depletion of IgG from nasal polyp tissue homogenates resulted in an increase in IgE-facilitated allergen binding to B cells but also enhanced FcεRI-mediated allergen-driven basophil activation and histamine release (145). A similar response was observed in relation to SE-IgEs (145). In fact, IgG repertoires share extensively the same antigen targets with IgE repertoires in both allergic and non-allergic subjects and nasal polyps are characterized by abundant clonally related IgG- and IgE- secreting plasma cells (145).

Allergen-specific IgGs are competing with IgEs for allergen binding, thereby decreasing the allergen-induced effector cell activation (3). This suggests that augmenting the allergen specific IgG/IgE ratio could be effective in preventing immediate hypersensitivity responses and reducing allergic symptoms (3, 146).

## Conclusions and Future Perspectives

In this review we summarized current data concerning the role of IgE in COPD and asthma (Figure 3). It appears that IgE production plays an important role in the development of both diseases. Interestingly, allergic sensitization and viral infections in infancy have been associated with a higher prevalence of asthma later in life, as well as with the most important determinant for COPD, namely the level of airflow limitation in late adulthood. Anti-IgE therapy (omalizumab) prevents viral exacerbations of asthma and COPD with asthmatic characteristics. Moreover, allergen-specific IgGs are competing with IgEs for allergen binding and augmenting the allergen specific IgG/IgE ratio could be effective in preventing immediate hypersensitivity responses and reducing allergic symptoms. Additionally, the suppression of microRNA-21-5p may present a therapeutic target to reduce airway wall remodeling induced by IgE. Further studies that will analyze the allergic profile of COPD patients in association with lung function, disease severity, outcome, exacerbations and airway remodeling are warranted, in order to examine a potential use of anti-IgE treatment for COPD patients.

## Author Contributions

MK and DS: conception of the review topic. MK, EP, AG, and DS: literature review, writing of the manuscript, finalization of the manuscript, and approval of the submitted article. All authors contributed to the article and approved the submitted version.

## Conflict of Interest

The authors declare that the research was conducted in the absence of any commercial or financial relationships that could be construed as a potential conflict of interest.

## Publisher's Note

All claims expressed in this article are solely those of the authors and do not necessarily represent those of their affiliated organizations, or those of the publisher, the editors and the reviewers. Any product that may be evaluated in this article, or claim that may be made by its manufacturer, is not guaranteed or endorsed by the publisher.
